# Job Demands and Resources Perceived by Dentists in a Digital Dental Workplace and Perceived Effects on Job Satisfaction and Stress: A Qualitative Study

**DOI:** 10.3390/clinpract15050092

**Published:** 2025-05-12

**Authors:** Julia Sofie Gebhardt, Volker Harth, David A. Groneberg, Stefanie Mache

**Affiliations:** 1 Institute for Occupational and Maritime Medicine (ZfAM), University Medical Center Hamburg-Eppendorf (UKE), Seewartenstraße 10, 20459 Hamburg, Germanyharth@uke.de (V.H.); 2 Institute of Occupational, Social and Environmental Medicine, Goethe University, 60596 Frankfurt, Germany; groneberg@med.uni-frankfurt.de

**Keywords:** digitisation, dentistry, dental workplace, dentists, job demands, job resources, stress perception, medical staff, practices

## Abstract

**Background:** Digitalisation is becoming increasingly integrated into the field of dentistry; therefore, it is crucial to understand both the challenges it introduces and the opportunities it provides. By doing so, the research will offer insights into how digital tools can affect the work environment and contribute to the overall well-being and performance of dental professionals. **Objectives:** The present study aims to explore how dentists perceive the demands and resources within a digitalised dental practice. **Methods:** The present study adopted a qualitative design, incorporating guideline-based interviews. A total of 30 interviews were conducted with dentists from various German dental practices, with a focus on key topics such as job demands, job resources, digital stress factors, job satisfaction, and support needs in the context of digital dentistry. The interview data were analysed using qualitative content analysis. **Results:** The findings highlight that digital systems in dental practices offer benefits such as reduced errors and time savings, but also pose challenges, especially for less experienced users. While they improve efficiency, precision, and professional development, they can also lead to negative effects like dependence on technology, loss of manual skills, technical failures, and increased stress, particularly during the adaptation phase. These results suggest that successful integration of digital technologies requires adequate support to overcome initial learning curves and ensure long-term benefits. **Conclusions:** The results of the study underline the importance of effective implementation, comprehensive staff training, and technological reliability to maximise the benefits of digital tools and minimise their drawbacks. Maintaining a balance between digital stressors and resources is crucial to promoting a healthy work environment. Future research should focus on the long-term effects of training programmes and the integration of digital technologies into dental practices to increase their effectiveness in terms of job satisfaction and reduce potential risks.

## 1. Introduction

Dentistry has made significant progress in recent years through the integration of new digital technologies [1,2,3]. These innovations have not only improved the quality of patient care but have also significantly changed the working practices of dentists. The most important digital developments include:

1. Digital imaging and 3D X-rays

Digital imaging, in particular, cone beam computed tomography (CBCT), has revolutionised the diagnosis and planning of treatments. It enables precise 3D visualisation of dental anatomy, which is particularly beneficial in implantology, oral surgery, and the diagnosis of dental diseases. These technologies help to reduce treatment errors and optimise treatment planning [4].

2. Computer-aided design/computer-aided manufacturing (CAD/CAM) technology

CAD/CAM systems are increasingly used in dental practices to design and manufacture restorations such as crowns, bridges, and veneers directly on site. These technologies improve speed and precision, enhancing both patient comfort and practice efficiency. Studies have shown that CAD/CAM can significantly reduce treatment time and improve fit accuracy compared to conventional methods [5,6,7].

3. Teledentistry is gaining importance as a tool for remote consultation and diagnosis, especially in underserved or rural areas. It offers improved access to care and can help reduce administrative workload in dental practices. Studies have shown its effectiveness in preventive care, triaging, and follow-up consultations [8]. However, limitations remain—particularly regarding the need for stable digital infrastructure and concerns about the diagnostic accuracy compared to in-person visits [9,10]. Ensuring data security and maintaining patient trust are also critical challenges for broader implementation.

4. Robot-assisted surgery and AIRobot-assisted systems are becoming more prevalent in areas like implantology, providing enhanced precision in implant placement and reducing human error. AI is also increasingly used in diagnostics, such as detecting caries and periodontal diseases from X-ray images. These technologies hold significant potential for improving treatment quality, but they also require substantial training and adjustments to practice workflows [11,12]. While robot-assisted systems enhance precision, some studies suggest challenges related to system integration and initial setup costs [13]. AI, though highly promising, also requires continuous validation to ensure diagnostic accuracy [3,14].

The advent of digital dentistry has precipitated a paradigm shift in work requirements and resources. For instance, digital radiographs facilitate expedited diagnosis and more exact treatment, whereas computer-aided planning and navigation systems facilitate the performance of intricate procedures [15,16]. Digital radiography and computer-aided planning and navigation systems not only enhance efficiency but can also augment professional satisfaction by enabling dentists to dedicate more time to patient care and less to administrative tasks. Nevertheless, digital technologies can also impose novel demands on dentists, particularly in regard to the digital competencies necessitated and the capacity to adapt to the relentless advancement of technological innovations [17]. While digital technologies are often associated with increased efficiency, recent studies suggest that their integration into dental workflows can, in many cases, lead to increased time spent on computer-based tasks, including documentation, planning, and software management [18]. This may result in less direct chair-side time rather than more. Awad et al. (2021) demonstrated that health care providers using fully integrated digital systems reported a significant increase in administrative workload, which in turn affected their available time for patient interactions [19]. Hence, the impact of digitalisation on clinical time allocation appears to depend on both the type of technology and the extent of its integration into practice [20].The utilisation of novel technologies can impose an additional cognitive burden on dentists, who are required to continually update their skills in order to remain proficient in the face of evolving demands. For example, the integration of digital impression systems, CAD/CAM technology, and artificial intelligence-assisted diagnostic tools demands not only technical competence but also the ability to critically evaluate digital outputs, interpret software suggestions, and troubleshoot workflow disruptions. These tasks require sustained attention, rapid adaptation to software updates, and decision-making under time constraints, all of which contribute to increased cognitive load [21,22]. Furthermore, the occurrence of technological malfunctions, in conjunction with the perpetual necessity for system maintenance, has the potential to engender feelings of stress and frustration among dentists [23]. The paucity of training in the utilisation of these technologies may give rise to feelings of anxiety and a concomitant diminution in job satisfaction, which, in turn, may precipitate an increased risk of burnout [23,24].

Conversely, the implementation of novel technologies in the absence of adequate guidance and communication has been demonstrated to give rise to ambiguity and discord within the team. This may stem from unclear role definitions, inconsistent understanding of the technology’s application, and unequal levels of training among team members. For instance, dental assistants may be expected to interact with digital imaging systems without being given formal instruction, leading to inefficiencies and potential errors. Moreover, when responsibilities for using or maintaining new systems are not clearly allocated, misunderstandings and frustration can arise, ultimately affecting team cohesion and workflow efficiency. As such, effective team-based training and open channels of communication are essential to ensure a smooth integration of new technologies into clinical routines [25,26,27].

### 1.1. Theoretical Background

Concurrently, the aforementioned technologies have introduced novel challenges for dentists, which can be elucidated through the lens of the job demands–resources (JD-R) theory.

The JD-R model, originally developed by Bakker and Demerouti (2007), posits that job demands and job resources are the primary determinants of employee well-being, motivation, and performance [28]. Job demands, which have been identified as significant contributors to stress and burnout, include high work intensity, emotional strain, and time pressure. Conversely, job resources, including social support, professional autonomy, and training opportunities, have been identified as crucial for coping with these demands and promoting well-being [28,29].

The JD-R model forms the theoretical framework of many empirical studies that are specifically relevant to medical practice and can now also be transferred to the context of dental practice. It supports investigating the effects of job demands and resources on outcomes such as burnout, job satisfaction, and quality of care.

The perception of job demands and job resources in a digital working environment may prove to be a significant factor influencing how dentists experience the introduction of new technologies. The manner in which these demands and resources are managed is closely associated with well-being and professional performance. A substantial body of research has demonstrated that an elevated perception of job demands in the absence of adequate resources is a significant predictor of increased stress and burnout [30,31]. For instance, a study by Gómez-Polo et al. (2022) reported that nearly 45% of dental professionals who perceived high workload without corresponding support resources exhibited clinically relevant levels of burnout symptoms [32]. Conversely, the perception of sufficient work resources, such as the availability of further training opportunities or technical support, has been shown to facilitate more effective stress management and enhance job satisfaction [30].

### 1.2. The Impact of Digital Stressors and Resources on Job Satisfaction and Perceived Stress: Current State of Research

The utilisation of digital resources, encompassing user-friendly software, accurate diagnostic tools, and the automation of routine tasks, may have the potential to reduce dentists’ workload and enhance satisfaction by virtue of saving time and minimising errors [33]. Furthermore, research has demonstrated that access to digital continuing education resources enhances professional competence and confidence, which in turn fosters professional satisfaction [30]. Conversely, digital stressors, including technological malfunctions, constant software updates, and the necessity for continuous training, have the potential to elevate the perception of stress [23].

The perception of digital stressors is of particular relevance, as they not only affect psychological well-being but may also have a negative impact on job performance and overall job satisfaction. In a study conducted by Bernburg et al. (2025), it was discovered that dentists who experience feelings of being overwhelmed or who encounter difficulties in utilising digital technologies tend to report higher levels of stress [34]. This may be further compounded by inadequate implementation of digital systems or an absence of training and support, which can intensify feelings of being overwhelmed and perceived stress.

### 1.3. Study Aim

The aim of this study is to investigate the perceived job demands and resources of dentists in a digitalised dental practice. Given the increasing integration of digital technologies into dentistry, it is crucial to understand the challenges and opportunities associated with this. The research aims to shed light on how digital tools influence the work environment and contribute to dentists’ job satisfaction and well-being.

The study answers two key research questions:

1. What job demands and jobs resources do dentists perceive in digitalised dental practice? This question examines the specific requirements and resources in dealing with digital technologies such as electronic health records, digital imaging, and CAD/CAM systems.
2. What associations exist between these work requirements and work-related outcomes such as job satisfaction? Research is being conducted into how perceived demands and resources influence perceived job satisfaction, stress levels, and mental health.

## 2. Materials and Methods

### 2.1. Study Design

In consideration of the subject matter and the research question, the qualitative method of guideline-based interviews was selected for this study. A qualitative research design is required to ensure sensitive recording and differentiate between different topics, such as the specific influence of normative assumptions, subjective theories, or the reconstruction of forms of everyday knowledge [35,36]. The study involved conducting 30 interviews in a personal meeting setting. In order to achieve and assess adequate reporting quality, the COREQ checklist [37] was used as the basis for the evaluation of the expert interviews.

### 2.2. Study Sample and Recruitment of Participants

The target group of the research project is dentists in Germany. In order to comply with the idea of a target group with few selective characteristics, the interview partners were recruited from a variety of dental practices throughout Germany. The study participants were recruited via the snowball procedure within the research community. Furthermore, the gatekeeper principle was employed as an additional recruitment strategy. In this approach, key personnel within the dental practices assume the role of initiators, actively seeking to engage employees in interviews. Consequently, the dental care services were contacted via e-mail and/or telephone. The attempt to form a sample that corresponds to the heterogeneity of the field is in accordance with the quality criterion of external validity in qualitative research [38,39].

In order to obtain a representative and relevant sample for this study, a set of inclusion criteria was established to guide participant selection. The criteria were designed to ensure that all participants possessed sufficient professional background and practical experience with digital technologies in dental practice. Eligible participants were required to meet all of the following criteria:

- Work Activity: Only licensed dentists were considered for inclusion.
- Work Environment: Participants had to be actively working in a dental practice, a multi-care centre, or a dental clinic.
- Employment Relationship: Both employed dentists and those working in private practice were eligible.
- Digital Assistance Systems: Participants needed to have both experience with and current use of digital technologies relevant to dentistry (e.g., digital radiography, intraoral scanning, digital documentation, or treatment planning systems).
- Application Experience: A minimum of six months of continuous application experience with digital systems in clinical practice was required.
- Language Skills: As the study was conducted in German, only German-speaking participants were included to ensure consistent understanding of the survey content and terminology.

These criteria helped ensure a consistent and knowledgeable participant pool, which was essential for obtaining valid insights regarding the use and perception of digital assistance systems in dental care settings.

Participation in the study was voluntary for the dentists. Prior to the commencement of the interviews, each participant was requested to sign a declaration of informed consent regarding performance and recording. All interviewees were deemed to have the capacity to comprehend and consent to the study requirements and thus were provided with written informed consent.

### 2.3. Interview Guidelines and Data Collection

A semi-structured interview guide was designed within the general framework of the empirical and theoretical background. The interview guide can be found in Supplement S1.

Questions on the following categories were asked, as presented in Table 1.

The interviews took place between August 2023 and September 2024. The interview guide was initially reviewed by four scientists with expertise in qualitative research methodology and was subsequently piloted with six dentists to receive feedback and to assess the duration of the interview and the comprehensibility of the questions. Their input was invaluable in identifying key areas of interest, such as the practical challenges of digital technology integration, its impact on workflow efficiency, and its influence on patient care. This collaborative process ensured that the interview guide addressed both the technical and the human factors involved in digital dentistry, thereby enhancing its content validity and relevance to the study’s objectives. The interview partners fulfilled all the inclusion criteria described above. The interviews were recorded with an audio device and then transcribed [38]. The interviews were conducted in German and averaged 40 min in length. The interviews were then transcribed using the audio recordings, which formed the basis for the data evaluation.

### 2.4. Data Analysis

As the evaluation methodology of the data material within the framework of this research project, the qualitative content analysis according to Kuckartz and Rädiker (2020) was chosen [40]. Based on the research question, the first step was to work on the text using transcriptions of the interviews recorded on audio. The transcript should give the researchers and readers the best possible impression of the interview, but too many details lead to poor readability. The transcription was carried out using the f4transcript programme, a software developed by F4 Development, which is based in Germany.

The formation of categories was guided by a set of predefined criteria and the existing body of literature, with the JD-R model providing the theoretical framework. Given the research project’s objective to examine a theoretical framework—to which the aforementioned guidelines also refer—the categorisation process could be initiated prior to the analysis of the collected data.

The qualitative content analysis was conducted in an iterative process in which categories were initially developed deductively on the basis of the theoretical framework and the research questions and then inductively expanded and specified in the course of the analysis (see coding system in Supplement S2). A priori categorisation (deductive categorisation) was employed in phase 1. However, following the initial categorisation, the project also underwent an inductive categorisation process (phase 2) [40]. This approach was adopted in response to the emergence of main categories and sub-categories during the evaluation of the data material and based on the initial findings. The data analysis was conducted using MAXQDA 2020 (VERBI Software, 2019, VERBI GmbH, Berlin, Germany). To ensure the highest standards in the evaluation of the data, the category system underwent successive modifications throughout the analysis process (see Supplement S2).

## 3. Results

### 3.1. Sociodemographic and Occupational Characteristics of the Study Participants

The demographic composition of the sample was as follows: The participating interviewees were 24 to 59 years old, 60% of the subjects were female, and 90% of them were engaged in full-time employment for a minimum of 80% of a standard working week. The range of work experience varied from a minimum of seven months to a maximum of 20 years. A total of 56% of the interviewees were practice owners. Further details concerning the occupational and sociodemographic characteristics of the participants are provided in the Table 2.

### 3.2. Job Demands and Resources Perceived by the Dentists in a Digital Dental Workplace

In accordance with the JD-R model, the participants of the interviews were asked to report on their perceptions of the job demands and resources present in a digital dental workplace. An overview of the different factors is provided in the subsequent sections, with further elaboration in the ensuing Table 3. The ensuing sections provide detailed explanations of the results. All quotations can be found in Supplement S3.

Dentists reported diverse experiences regarding job demands in digital dentistry, particularly in terms of workload, delegation, error susceptibility, and acceleration. While digital tools were often associated with reduced workload and faster processes, some practitioners perceived increased or unchanged workload levels.

Susceptibility to errors

A significant number of interviewees expressed a perception of reduced susceptibility to errors through the use of digital technologies. Traditional analogue procedures, such as alginate impressions or manual model fabrication, were perceived as more error-prone due to their numerous manual steps and greater dependency on material handling. In contrast, digital tools like intraoral scanners, CAD/CAM systems, and digital radiography were described as more accurate, reproducible, and less dependent on user variability.

In particular, participants emphasised the advantages of being able to visualise and correct errors directly in the digital workflow—for example, by selectively rescanning areas or adjusting preparation margins independently. One dentist noted, “*With digital impressions, we have reduced both the error rate and the working time for dental procedures by 50%*”. (P5, male, 30–39)

Digital radiography was also highlighted for its improved resolution, faster availability, and integration with diagnostic software that supports measurement and interpretation. In this context, participants described a greater sense of diagnostic precision and safety. Similarly, the use of 3D imaging (DVT) and guided surgery was considered to reduce clinical risks, especially in implantology.

Moreover, digital documentation systems were perceived as reducing errors in patient records due to built-in plausibility checks and the ability to edit entries flexibly using text modules. Several dentists also noted that improved structure and standardisation in digital workflows contributed to fewer avoidable mistakes during treatment.

While many respondents highlighted the error-reducing potential of digital tools, a smaller number also noted that reliance on technology brings new challenges, such as technical malfunctions or the need for additional training to effectively utilise software features. Nevertheless, the overall trend indicates a clear perception of improved accuracy and reliability through digitalisation.

Increased susceptibility to errors

While most participants perceived digital systems as reducing error rates, several interviewees also reported situations in which digital tools could introduce new sources of error. These were often attributed to unfamiliarity with the technology or insufficient training. In particular, inexperienced users were seen as more prone to mistakes, especially when lacking a thorough understanding of system functionalities or workflows. As one dentist noted,

“*Some use it to achieve maximum success, while others use it but struggle with it. And then, of course, you also have errors*”.

(P9, female, 50–59)

Moreover, some participants described limitations in the reliability of digital implant planning. Discrepancies between virtual planning and clinical conditions, such as changes in bone structure due to resorption, were reported as potential risk factors. These deviations made it necessary to verify digital plans intraoperatively rather than relying solely on surgical guides.

Additionally, digital workflows were seen as vulnerable to data-related errors, including the selection or transmission of incorrect files. For example, one participant reported receiving an X-ray from a referring provider that did not correspond to the patient’s actual condition. Scheduling and planning errors, such as incorrect appointment durations or overlooked software alerts, were also mentioned.

These findings suggest that while digital systems offer considerable benefits, they may also create new types of error risks if not carefully integrated into clinical routines. Proper training, verification steps, and system familiarity appear crucial to minimising these issues.

Time Savings and Increased Efficiency

A central benefit consistently reported by the participants was the time-saving potential of digital technologies. Digital impressions, CAD/CAM systems, and digital radiography were perceived as significantly accelerating clinical workflows—particularly in implantology and prosthodontics—by enabling faster production of restorations, improved diagnostic access, and more efficient information flow within the practice.

Participants highlighted that procedures such as intraoral scanning or digital chairside workflows (e.g., with CEREC systems) not only reduced the duration of patient visits but also allowed for same-day treatment in certain cases. The immediate availability of digital radiographs and their direct integration into patient files further streamlined diagnostics and communication with colleagues or external clinics. This was seen as especially advantageous in emergency situations or when referring patients.

Several interviewees emphasised that digital systems contributed to overall higher productivity, noting that more treatments could be completed in less time, while simultaneously improving the quality and consistency of clinical outcomes. Additionally, the delegation of tasks—such as scanning or preliminary documentation—to trained dental assistants was cited as a key factor in increasing flexibility and patient throughput.

Other time-saving aspects included the use of text modules in digital documentation, which reduced writing time, as well as faster access to patient histories via electronic health records. The option to send forms and treatment plans digitally before appointments was seen as further enhancing efficiency and workflow organisation.

Overall, participants described digitalisation as a substantial facilitator of clinical efficiency, particularly when digital tools were well integrated into the daily routines of the dental team.

No Time Saving and Technical Limitations

Despite the frequently reported efficiency gains, several participants emphasised that digital technologies do not inherently lead to time savings. In some situations, technical difficulties—such as software errors, device malfunctions, or connectivity issues—can cause significant delays and frustration. One participant noted,

“*What’s frustrating is that when it doesn’t work, you end up spending more time than with conventional methods*”.

(P9, female, age 50–59)

Tasks such as the digital design and milling of prostheses may also require substantial time resources, especially when complex restorations are involved. Additionally, scanning procedures were occasionally described as time-intensive, particularly in cases where intraoral conditions (e.g., moisture or patient movement) complicated the process.

“*I can also take fifteen minutes to scan if I’m having trouble getting a certain spot and have to keep drying it out and checking*”.

(P2, female, age 20–29)

Moreover, the initial setup time for digital systems—such as booting computers or navigating software interfaces—was compared unfavourably to the immediate access to paper-based records. These findings suggest that time efficiency strongly depends on the reliability of the technology, the user’s experience, and the specific clinical context.

Quality and Aesthetics

Many participants highlighted improvements in diagnostic precision and treatment quality due to digital technologies. Digital X-rays and DVT scans were frequently praised for their enhanced resolution and greater diagnostic depth, particularly in endodontics and implantology.

“*X-rays are an absolute must, and digital images are, of course, much more valuable for assessment than an analogue image*”.

(P4, female, age 30–39)

In the field of prosthetics, CAD/CAM-manufactured restorations and splints were described as more precise and aesthetically pleasing than their conventional counterparts. Participants emphasised the superior fit, material quality, and natural appearance of digitally fabricated restorations, especially when using multilayer ceramics.

“*The restorations not only fit better, but they also look more aesthetic [...] It’s much better than what conventional dentistry can achieve in certain areas*”.

(P9, female, age 50–59)

Additional advantages mentioned include improved hygiene due to paperless documentation and higher precision through digital facebows and intraoral scanning. While some processes (e.g., fabrication of surgical guides) may take more time, the trade-off was often seen as justified by increased safety and patient benefit.

### 3.3. Adverse Effects of Digital Assistance Systems on Job Satisfaction

While digital assistance systems can greatly enhance the efficiency of medical and dental practices, they also introduce challenges that can negatively impact job satisfaction. The following explores several negative effects (as presented in Table 4), supported by insights and reflections from dental professionals.

Dependency on Technology

Some participants expressed concerns about becoming overly dependent on digital systems, which can increase vulnerability if technology fails. This dependency was seen as potentially limiting professional autonomy and control.

“*Dependence is definitely growing. […] the more technology there is, the more vulnerable the entire construct becomes*”.

(P1, male, age 30–39)

Decline in Personal Skills

The use of digital aids like drilling templates may erode manual skills and reduce practitioners’ confidence, particularly in complex procedures. Over time, reliance on digital support can diminish critical thinking and clinical autonomy.

“*I think at some point I would no longer trust myself to do it without the template […] with complicated things, I could imagine that you become more cautious and less confident*”.

(P7, female, age 30–39)

Rapid Technological Advancement

The fast pace of innovation in digital dentistry can overwhelm practitioners. Constant updates and lack of industry support were cited as barriers to long-term integration.

“*We wanted to lease it […] but the industry did not provide that*”.

(P10, female, age 50–59)

Lack of Control Over Technology

Participants voiced fears about digital tools becoming autonomous or externally controlled, undermining professional independence and creating a sense of helplessness.

“*It scares me what is all controllable and how interventions can be made in my processes, making me dependent*”.

(P10, female, age 50–59)

General Workload and Bureaucracy

While digital assistance systems offer potential efficiency gains, they are often embedded within an already demanding work environment. According to participants, the actual stress factors stem less from digitalisation itself and more from the accumulation of responsibilities, administrative burdens, and practice management tasks. The integration of new technologies can thus contribute to an increased sense of overload, not necessarily due to the tools themselves, but because they add to an already high workload.

“*It’s more from all the stress or the overall burden of the work, running the practice, and the bureaucracy—there’s a lot more to it. But I don’t think it’s directly related to the digital transformation*”.

(P7, female, age 30–39)

### 3.4. Positive Effects of Digital Assistance Systems on Job Satisfaction

Apart from the challenges that digital technologies bring, many users also experience a positive impact on job satisfaction by simplifying tasks, increasing motivation, and fostering professional growth. The following explores several positive effects, supported by insights and reflections from dental professionals (see also Table 5).

Work Simplification

A key reason for the positive effect of digital assistance systems on job satisfaction lies in the perceived simplification of daily tasks. Digital technologies make routine work easier, enhance efficiency, and reduce physical strain—contributing to overall well-being in the long term.

“*I really believe that it will contribute to my physical well-being in the long run, because it will make everything much easier for us*”.

(P2, female, age 20–29)

This increased speed and precision enable dental teams to accomplish more in less time. At the same time, unnecessary steps are eliminated, and workflows are streamlined, making daily routines more manageable. One participant shared how this impacts her day-to-day work:

“*What motivates me is the simplicity […]. I can take the image, see it immediately, and keep working—that’s what I perceive positively*”.

(P7, female, age 30–39)

In addition to practical relief, some interviewees emphasised that well-integrated digital systems support a smooth, uninterrupted workflow. When everything functions properly, it creates a sense of “flow”, which greatly enhances job satisfaction.

Motivation Enhancement

The introduction of digital systems has had a motivational impact on users, sparking enthusiasm and creativity. The novelty of these digital technologies drives engagement, with participants expressing a sense of excitement.

“*The intraoral scanner is definitely the most exciting of all the things, but overall, I can say I’m highly motivated to keep going with it*”.

(P3, male, age 50–59)

For some, the ability to approach challenges creatively and explore new solutions adds to their motivation, making the work more dynamic.

“*Yes, it’s definitely something new. And in general, something new is potentially also something that makes you more motivated*”.

(P6, female, age 20–29)

Sense of Fulfilment

Many participants find great satisfaction in using digital tools, as these allow them to produce high-quality work. The ability to create beautiful results brings a sense of pride and accomplishment.

“*It’s a lot more enjoyable because you can create really beautiful work with it. […] That’s something that’s satisfying and definitely increases job satisfaction*”.

(P4, female, age 30–39)

For others, seeing their work fit perfectly—like placing implants or crafting crowns—further enhances their sense of fulfilment.

“*Or I place implants in three dimensions, and afterwards they fit perfectly, making the patient happy—and me too*”.

(P9, female, age 50–59)

Physical and Mental Health Benefits

Digital tools contribute to better physical health by reducing strain, particularly with tasks like X-ray handling or using intraoral scanners. Participants recognise the long-term health benefits these technologies provide.

“*And with the X-rays, of course, it’s much better health-wise for the assistants. They no longer have to handle those physical images or operate any machines in a darkroom*”.

(P2, female, age 20–29)

Rather than causing burnout, these systems support the work environment and alleviate stress.

“*Absolutely not. I can’t see any reason why I would get burnout because of digitalisation. It actually helps me more*”.

(P9, female, age 50–59)

Skill Improvement through Experience

Using digital tools allows professionals to continuously refine their skills, which increases both job satisfaction and confidence. The learning process is seen as part of the journey to mastery.

“*Exactly, it will definitely get better at some point. That you’ll be in sync with the feeling, I would say*”.

(P7, female, age 30–39)

The process of learning through experience, including occasional mistakes, further enhances personal growth.

“*No, I think it’s important to sometimes make mistakes or not do something perfectly, so that you have the drive to improve next time*”.

(P2, female, age 20–29)

Increased Economic Returns

Participants also noted that digital tools lead to increased productivity, which has economic benefits that further reinforce job satisfaction.

“*Yes, but when you eventually realise that everything is working well and fitting perfectly, it’s a wonderful feeling. Especially when you know that you created it yourself*”.

(P8, female, age 20–29)

Economic gains were tied to improvements in efficiency, which in turn boosted job satisfaction.

“*I think that overall it will get better, especially if many of the things I’m hoping for now work well. […] Then I believe it will bring ease and, with that, greater satisfaction*”.

(P7, female, age 30–39)

### 3.5. Effects of Digital Assistance Systems on Stress Experience

#### 3.5.1. Negative Effects on Stress Experience

Digitalisation in dental practices introduces several factors that increase stress (see Table 6).

Digitalisation in dental practices has introduced several stressors that affect both dentists and staff. A major source of stress is uncontrollable technical malfunctions, such as software crashes, leading to time loss and increased pressure. Participants highlighted the frustration of dealing with these disruptions.

“*It’s annoying that when it doesn’t work, you end up spending more time than usual*”.

(P9, female, age 50–59)

“*That causes stress. So, when it doesn’t work. When it does work, it’s great*”.

(P1, male, age 30–39)

The growing dependency on digital systems also amplifies stress, as participants feel a sense of reduced control over their work. They emphasised how external issues like device failures can disrupt workflows and create anxiety.

“*The only thing […] is when you have external disturbances that I cannot control. For example, if the scanner collapses in the middle of a scan for some reason. Then you think to yourself, ‘I didn’t even do anything.’ And then you have to start over again. That’s stressful*”.

(P9, female, age 50–59)

Some participants also reported a lack of trust in technology, which adds to their anxiety, especially when the systems are not functioning as expected.

“*If I had negative experiences and faced setbacks every day—if things didn’t fit, if it didn’t look nice, if the height wasn’t right, if the shape wasn’t right—then that would be something else*”.

(P9, female, age 50–59)

Financial pressures, such as the high acquisition costs of digital systems, also contribute to stress. The uncertainty around new workflows, data security, and system integration adds psychological pressure.

“*It’s perhaps important to mention that it’s a higher initial investment. And that cost pressure is definitely present at the beginning because you don’t really know how it will turn out*”.

(P3, male, age 50–59)

Frustrations are further exacerbated when outdated or unreliable software causes inefficiencies, leading to both mental and time-related strain.

“*If the program constantly crashes, it’s naturally frustrating. And if you want to go home in the evening but then have to come back to the practice on the weekend because your record entries didn’t go through, that’s definitely a mental strain*”.

(P5, male, age 30–39)

Additionally, the need for more coordination and flexibility when using shared tools like scanners adds complexity to workflows.

“*When I want to use the scanner, and someone else is using it, that already impacts my work because I can’t move forward*”.

(P2, female, age 20–29)

Cybersecurity concerns, such as the risk of hacker attacks, were also mentioned as stress-inducing, as digital systems become more vulnerable.

“*The worst thing that could happen, of course, would be a hacker attack or something like that. You’re obviously very vulnerable to that*”.

(P2, female, age 20–29)

Finally, the pressure to achieve perfection and constantly optimise results leads to stress, particularly for younger dentists who may feel the burden of performance comparisons.

“*I often worry about whether it was perfect, and if it wasn’t, what caused it and what I could have done better*”.

(P8, female, age 20–29)

#### 3.5.2. Positive Effects on Stress Experience

On the other hand, digitalisation in dental practices also offers several factors that reduce stress levels. Many practitioners report that they do not experience increased stress when using digital tools.

“*Fatigue and stress aren’t an issue for me at all. So, more positive overall. And if there’s something I don’t know, then of course you’re a bit behind. You have to look it up or ask someone. But otherwise, it’s entirely positive*”.

(P9, female, age 50–59)

“*So, it definitely has a psychologically positive effect, you could say*”.

(participant 4, female, age 30–39)

For some practitioners, the flexibility and new possibilities provided by digital tools reduce stress and foster excitement and curiosity about exploring new technologies. Digital systems, such as customised dental prosthetics production, allow for a more innovative and personalised approach.

“*Of course, it’s a lot more fun. You can also do so much more with digitalisation. Especially in the lab, we have completely different possibilities with digital work, like creating constructions on implants*”.

(P4, female, age 30–39)

Digital tools also promote continuous learning and adaptation, keeping employees intellectually engaged. Many see digitalisation as a stimulating challenge that motivates them to stay updated with technological progress.

“*Yes, digital work absolutely motivates me, and I’m definitely willing to acquire new skills to push the technical possibilities even further*”.

(P5, male, age 30–39)

“*Mentally, at least, the constant contact with digital media forces you to stay up-to-date. So, in that sense, it’s definitely progress*”.

(P1, male, age 30–39)

For some, digital systems also help support their ambition to excel professionally, leading to greater satisfaction in meeting high personal and professional standards.

“*Yes, I have the ambition to continue developing myself. You really have to make sure that it works and that it is definitely improved to be effective and good*”.

(P4, female, age 30–39)

The precision and efficiency of digital workflows further reduce stress, especially by eliminating the need for extensive manual adjustments. Participants noted that better-fitting prosthetics lead to more patient interaction and less rework, creating a greater sense of control.

“*And I definitely notice it, thanks to the good fit and advanced technology. The collaboration with the technician has improved significantly, which adds to my satisfaction*”.

(P3, male, age 50–59)

Additionally, digital tools like implant drilling guides offer greater safety and reliability, reducing stress by minimising errors and increasing peace of mind.

“*When placing an implant, it fits exactly as planned, and that’s a great feeling. The peace of mind knowing you won’t be losing sleep over possibly injuring a nerve is very reassuring*”.

(P7, female, age 30–39)

Positive feedback from patients also boosts motivation, encouraging practitioners to refine their skills further.

“*I’m very technically inclined and have a strong affinity for software by nature. I definitely find it much more appealing to do a 3D scan, as it’s such an interesting process compared to taking an impression*”.

(P5, male, age 30–39)

Lastly, digital tools foster collaboration and shared learning, creating a supportive environment where practitioners can learn from one another’s experiences.

“*It means it’s also a source for sharing experiences. Discussing patient cases becomes easier through digitalisation, as everyone can access the information at any time*”.

(P8, female, age 20–29)

## 4. Discussion

The present study results demonstrate a broad spectrum of experiences among dentists concerning digital assistance systems, which have been shown to exert a dual impact on workload, with both positive and negative effects. These effects include an increased susceptibility to errors, as well as a shift in task delegation and the acceleration of work processes.

### 4.1. Job Demands and Resources in the Digitalised Dental Practice

#### 4.1.1. Reducing Susceptibility to Errors Through Digitalisation

This study emphasised the key advantage of digital assistance systems in reducing errors, a finding that has been reported by many of the surveyed dentists. The utilisation of digital impressions and radiographs was particularly noted for its ability to minimise errors in the digital workflow. These findings are consistent with the results of other studies that have demonstrated the accuracy and precision of digital technologies in dentistry. Albanchez-González et al. (2022) found that digital impressions and CAD/CAM technologies can significantly reduce the error rate in dental technology, especially compared to traditional methods such as the use of alginate impressions and plaster models [41]. Digital radiographs, which are instantly available and offer higher resolution, are also perceived by many practitioners as less prone to error [42]. Improved access to accurate diagnostic data, such as digital radiographs or CBCT scans, supports greater accuracy in diagnosis and treatment planning [43]. However, some participants also reported an increased susceptibility to errors, particularly due to ignorance or lack of experience in using digital systems. This mirrors the results of other studies, which show that the success of digitalisation in dentistry depends heavily on the experience of the user [43,44].

Studies highlighted that while digital technologies reduce human error, they also require dentists to adapt and refine their skills to optimise their performance [45,46]. Without proper training, practitioners might face technical failures or misinterpret data from digital systems, leading to new forms of errors, as reported by Waite et al. (2019), who found that novice users experienced higher error rates when using digital radiography compared to experienced users [47].

Furthermore, some contradictions arise from the integration of digital technologies into clinical practice. While digital systems are lauded for their precision and efficiency, the transition from traditional methods to digital workflows has been associated with initial learning curves and potential increases in stress and error rates. It is noted that early adoption of digital tools may lead to frustration and errors due to unfamiliarity with the systems, despite the long-term benefits of digitalisation in error reduction [48]. This contradicts the general notion that digital systems immediately lead to fewer errors, suggesting that the reduction in error susceptibility is a gradual process linked to practitioner experience and comfort with the new technology [49].

#### 4.1.2. Time Savings and Increased Efficiency

Another salient benefit frequently highlighted in the study pertains to the substantial time savings afforded by digital systems. Numerous participants identified the utilisation of CAD/CAM technologies, a hallmark of digitalisation, as a pivotal factor in accelerating workflows, a finding corroborated by studies demonstrating that digital workflows significantly reduce time expenditure in the field of dentistry. The utilisation of CEREC for the fabrication of restorations has been demonstrated in several studies to result in a substantial reduction in treatment time when compared to conventional methods [18,50]. The elimination of intermediate processes (e.g., casting models or awaiting laboratory results) and the reduction in fabrication steps enabled dental practices to function with greater efficiency and increase the number of patients treated [50]. These efficiencies are further supported by studies such as Mühlemann et al. (2019), who reported that CAD/CAM systems reduced chair time by approximately 40% for crown fabrication [51]. However, smaller practices may struggle with the integration of digital workflows due to limited resources or expertise, as pointed out by Ghaffari et al. (2022) [52].

#### 4.1.3. Delegation of Tasks

The delegation of tasks emerged as a salient theme in the interview results. The utilisation of digital systems was found to facilitate the efficient delegation of tasks, thereby reducing the workload burden on dentists. The study’s findings are consistent with those reported by several studies, which demonstrated that digital assistance systems in dental practices can optimise task distribution among staff, particularly through the use of intraoral scanners and digital radiographs, which can be delegated to assistants [53]. However, some participants also reported increased stress levels due to additional tasks and responsibilities associated with the introduction of new technologies. This finding is in line with the results of other studies indicating that the introduction of digital technologies can lead to some uncertainty and stress, especially in the initial phase of implementation [54].

#### 4.1.4. Dependence on User Experience

A further salient finding was that the success of digital technologies is contingent on the experience of dentists. A significant proportion of interviewees reported that the integration of digital technologies is accompanied by an initial learning curve, and that occasional failures may occur until practitioners are proficient with the systems. This assertion is corroborated by numerous studies that attest to the necessity of a substantial degree of technical proficiency for the effective utilisation of digital assistance systems in the dental domain. As highlighted in a study by van der Zande et al. (2020), the efficacy of digital systems exhibits significant variability, contingent on the user’s experience and training. Dentists with extensive experience of working with digital technologies report a significant improvement in efficiency and precision, while less experienced users tend to encounter difficulties [55].

Training and continuous support are crucial for effective digital integration, as highlighted by Matthews et al. [56]. Without proper support, dentists may experience stress and inefficiencies. However, Justice (2020) noted that varying levels of experience among practitioners can create inconsistencies in treatment outcomes and workflow efficiency within teams [57].

#### 4.1.5. Quality and Aesthetics in Digital Dentistry

The findings of this study demonstrate that digital technologies in dentistry offer substantial advantages in terms of accuracy and efficiency. In addition, they have the potential to enhance the quality and aesthetics of dental restorations and diagnostic procedures. Numerous participants reported higher precision and better assessability of digital radiographs compared to analogue, allowing for more accurate diagnosis, especially in endodontics. These findings are consistent with the extant literature that emphasises the advantages of digital imaging, especially in X-ray technology and CBCT (digital volume tomography). A review by Barai demonstrates that digital radiographs offer higher resolution, leading to more accurate diagnoses while reducing radiation exposure [58].

#### 4.1.6. Precision and Aesthetics of Digital Dental Restorations

The study demonstrated that digital technologies, such as CAD/CAM, have transformed the manufacturing process of dental restorations, particularly with regard to the precision and aesthetic quality of the restorations. Participants reported the high precision of digitally fabricated dental restorations, which exhibited superior fit and aesthetic appeal in comparison to conventionally fabricated crowns and bridges. These findings are consistent with those of previous studies that have emphasised the superior fit and aesthetics of CAD/CAM restorations. A review by Papadiochou (2018) confirmed that CAD/CAM restorations provide good marginal fit and improved aesthetics, especially due to the use of high-quality materials such as multi-layer ceramics [59].

Another advantage mentioned in this study is the aesthetic superiority of CAD/CAM-fabricated prostheses, which have natural colouration and better fit due to the use of multilayer ceramic materials. These findings are consistent with findings of a systematic review by Saravi et al., which emphasised the aesthetic advantages of digital prostheses while improving durability and functionality [60]. The utilisation of multilayer ceramics has been demonstrated to yield restorations that are both aesthetically pleasing and functionally optimal, thereby aligning with patients’ expectations regarding their dental restoration [60,61].

#### 4.1.7. Digital Diagnostics and Enhanced Treatment Outcomes

Digital technologies offer significant advantages, particularly in the field of diagnostics. The enhanced precision and clarity of contemporary X-ray sensors facilitates comprehensive diagnostic evaluations, a feature that is of particular relevance in the domains of endodontics and implant planning. As the study notes, CBCT scans offer greater diagnostic information than two-dimensional radiographs, providing a three-dimensional view that facilitates more accurate implant placement and superior assessment of bone volume. The benefits of CBCT in modern dentistry are further emphasised by numerous studies [62].

### 4.2. Positive Effects of Digital Assistance Systems on Job Satisfaction in the Dental Sector

The increasing integration of digital assistance systems in dental practices has far-reaching effects on the working methods and satisfaction of dental professionals. This discussion will analyse and discuss the positive effects of digital technologies on job satisfaction, based on the results of interviews with dental professionals, in the context of relevant scientific literature. The outcomes of this analysis include the simplification of work processes, increased motivation, enhanced fulfilment, health benefits, the improvement of skills through experience, and economic benefits.

The present study explores the ways in which digital systems have been shown to enhance job satisfaction, with a particular focus on the ways in which such systems can simplify work processes and increase efficiency. The respondents reported that the utilisation of digital systems has led to a substantial enhancement in the ease of their daily tasks, resulting in increased efficiency and precision. This finding aligns with the conclusions of studies demonstrating the efficacy of digital tools, such as intraoral scanners and digital radiographs, in enhancing efficiency within dental practices. As Dhillon mentioned (2024), the integration of digital technologies enables swifter diagnosis and treatment, thereby allowing dental practices to function with greater productivity without compromising the quality of care [63].

A quantitative study by van der Zande et al. (2020) showed that this enhanced efficiency subsequently leads to improved work organisation and a reduced workload, which in turn fosters greater job satisfaction [55]. The extant literature demonstrates a correlation between the simplification of work processes in dental practices through the use of digital tools and an increase in professional satisfaction [64]. A review by Ardila et al. (2023) found that the use of digital technologies, such as CAD/CAM systems for dental prosthesis production, reduces the workload for dentists and at the same time leads to higher productivity and quality [65].

In addition, the introduction of new technologies in practice is perceived by many professionals as a motivational factor. The interviewees further posit that working with digital tools not only facilitates precision but also fosters creative fulfilment, as it encourages the constant exploration of novel applications of these technologies.

This assertion is further substantiated by extant research. Studies showed that the use of innovative technologies in dentistry not only increases technical efficiency but also unleashes the creative potential of practitioners, leading to greater professional satisfaction and increased motivation [55,66].

The opportunity to develop and improve skills through the use of digital systems is another positive aspect. Van der Zande et al. (2018) emphasise that the continuous use of digital tools helps professionals expand their practical and technical skills, resulting in higher professional competence and increased job satisfaction [55]. This phenomenon is particularly salient among younger professionals who possess a high degree of digital proficiency and can continually enhance their skillset.

Regarding the economic advantages of digital technologies, Dhillon’s research indicates that their implementation may lead to enhanced job satisfaction and improved profitability of the practice. The efficiency gains and greater precision that digital technologies engender have been demonstrated to result in increased productivity and superior patient care, which in turn has a positive impact on the practice’s turnover and profitability [63].

Furthermore, the utilisation of digital tools has been observed to foster collaboration and knowledge exchange among colleagues, thereby strengthening their professional network and enhancing their sense of social support [67]. This positive impact of digital systems on collaboration and knowledge sharing has been emphasised by researchers, who have underscored the significance of collaborative platforms and case study sharing for job satisfaction and reduced stress levels in their respective studies.

### 4.3. Negative Effects of Digital Assistance Systems on Job Satisfaction

The advent of digital assistance systems within dental practices has profound ramifications for the modus operandi of professionals and their job satisfaction. Whilst digital tools have been shown to increase efficiency and improve quality of work in many cases, the results of the present study also demonstrate that digital systems are associated with various challenges that can have a negative impact on job satisfaction.

#### 4.3.1. Dependency on Digital Systems

The prevailing sentiment expressed by respondents in the interviews pertained to the escalating reliance on digital systems. A recurrent concern expressed by multiple interviewees pertained to the potential diminution of autonomy in their professional pursuits due to an overreliance on technology. These concerns are not unfounded, as several studies examine the psychological implications of increasing technological dependence [55,68]. According to Marsh (2022), reliance on technology can lead to what is known as “technology dependency”, where professionals feel that they are no longer able to perform their work effectively without the digital systems [69]. This dependency can lead to feelings of anxiety and a loss of self-confidence when technical problems occur [70]. Few studies also emphasise that the loss of control over digital systems can increase feelings of insecurity and frustration among users, which in turn affects job satisfaction [68].

#### 4.3.2. Decrease in Personal Skills and Clinical Competencies

A further recurrent theme pertained to the apprehension that the integration of digital tools might result in a diminution of crucial clinical competencies. For instance, the surgical guide system was cited in the interviews, illustrating how advancements in technology can enhance precision but concomitantly result in the deterioration of manual dexterity and critical thinking skills in more intricate scenarios. These concerns are corroborated by scientific research. Some research studies have highlighted that an excessive reliance on digital systems can result in the deterioration of practical skills, thereby impeding the capacity to address complex cases effectively [71]. This phenomenon may be of particular relevance in those professions that require manual dexterity and the capacity for rapid decision-making, such as dentistry. The erosion of these competencies has the potential to result in diminished job satisfaction and self-efficacy (see also Ref. [72]).

#### 4.3.3. Rapid Technological Progress and Lack of Adaptation

A recurrent theme in the discourse has been the pervasive sense of frustration with the accelerated rate of technological advancement, which has been accompanied by the concomitant challenge of maintaining pace with the incessant innovations that characterise this era. The rapid introduction of new digital tools and the necessity to update existing systems on a regular basis can result in stress and a sense of being overwhelmed. Schilbach et al. (2017) argued that employees may experience increased psychological strain if they are not adequately prepared for the changes [73]. A study by Bernburg et al. (2025) also discovered that dental professionals occasionally experience a sense of being overwhelmed when confronted with the rapid pace of technological advancement [34]. This phenomenon can result in diminished job satisfaction, particularly in instances where there is insufficient support to address these challenges. In contrast, the study by Teng et al. (2022) examined the impact of implementing digital dental technologies on job satisfaction and turnover intention among dental technicians in Taiwan. The findings indicate that greater acceptance of digital technologies is associated with higher levels of job satisfaction. However, the authors also point out that, despite a generally positive attitude toward digital tools, their impact on job satisfaction may be limited, particularly when high workloads and burnout risks are present simultaneously [74].

#### 4.3.4. Lack of Control over Digital Systems

The fourth issue pertains to a lack of control over digital systems. The absence of agency in the context of technology, a concern that emerged during the interviews, has the potential to engender feelings of frustration and erode confidence in digital systems. One of the participants in the interviews expressed concern about the possibility of digital devices influencing work in ways that professionals cannot control. This has been shown to engender feelings of insecurity and anxiety. These concerns have been addressed in the extant literature. Marsh et al. (2022) emphasise that the lack of control over digital systems and their malfunctions can lead to high stress levels among users, which negatively affects job satisfaction [69]. Further on, it is emphasised that a sense of control over digital technologies is paramount to fostering trust in these systems, and that a lack of control erodes confidence in technology [75].

#### 4.3.5. Organisational Problems

Another negative effect described in the interviews is the organisational stress that can arise from the introduction of digital systems. This phenomenon is further exacerbated by employee resistance to new technologies or inadequate integration of digital tools, potentially leading to an escalation in job dissatisfaction. These findings are consistent with those reported in studies investigating the impact of organisational change in the healthcare industry on job satisfaction. Tawfik et al. (2021) found that inadequate implementation of new technologies and the associated organisational challenges can lead to an increase in stress and frustration [76]. In a similar vein, Kumar et al. (2024) demonstrated the association between inadequate training and support from team members in the context of the introduction and utilisation of digital applications, which increases the risk of exhaustion [77].

#### 4.3.6. Increased Workload and Bureaucracy

Finally, the additional administrative and bureaucratic requirements associated with digitalisation were cited as a significant factor negatively impacting job satisfaction [78,79]. The present findings are consistent with the results of other studies, which emphasised that the implementation of digital systems in medical and dental practices can introduce further administrative burdens, thereby exacerbating the already high workload of professionals [80]. This has been shown to result in feelings of being overwhelmed and dissatisfaction, as the digital tools require additional system maintenance and administration tasks [44,78].

### 4.4. Effects of Digital Assistance Systems on Perceived Stress Levels in Dental Practices

The results demonstrate that the introduction of digital assistance systems in dental practices exerts a dual influence on the stress experienced by dentists, exhibiting both negative and positive effects. This dual perspective, encompassing both the potential stresses and benefits of digital systems, is reflected in the extant scientific literature and provides valuable insights into the complex interactions between technology and stress.

A frequently mentioned stress factor in the interviews is the unreliability of digital systems, in particular software errors and technical faults. These have been shown to cause loss of time and additional pressure [69]. This is perceived as particularly stressful by the interviewees, as time pressure is an essential part of dental work. The present findings are consistent with those of previous research, which demonstrated that technological failures and unpredictable sources of error have a significant impact on feelings of control and efficiency [76]. Marsh et al. (2022) also reported that reliance on digital systems combined with system failures can lead to increased workload and psychological stress as professionals find themselves helpless in the face of external disruptions [69].

Another salient stress factor that was mentioned in the interviews was the growing dependence on digital systems. These concerns have been previously articulated in research by Sanjeeva Kumar, who emphasised the deleterious impact of technological dependence on the experience of stress and feelings of insecurity [77].

### 4.5. Strengths and Limitations

The qualitative approach employed in this study provides in-depth insights into the experiences and perceptions of dental professionals regarding the integration of digital tools in their practices. By conducting interviews, the study was able to capture the nuanced views of practitioners who directly engage with digital technologies. The findings of this study are directly applicable to clinical practice, offering valuable insights into how digital tools influence stress levels, job satisfaction, and overall workflow. These insights can serve as a resource for dental professionals, administrators, and policymakers to optimise digital tool integration and address the challenges identified in the study. Participants came from various areas of dentistry, contributing to a broader perspective on the impact of digitalisation across different practice models. The study provides valuable insights that can be directly applied to optimising the use of digital tools in dental practices.

The small sample size limits the generalisability of the findings. A larger, more diverse sample would improve the validity of the results. Snowball sampling may have led to the inclusion of predominantly digitally savvy study participants. This may limit the generalisability of the findings to a broader population of dental professionals with varying levels of expertise in digital technologies.

While transcribed text provides a valuable written record of the interview, it is important to acknowledge that it may not fully capture the nuances of the interaction, such as tone of voice, body language, or other non-verbal cues. These elements can significantly contribute to the interpretation of the data, as they provide context that enhances the understanding of the interviewee’s emotions, emphasis, or underlying meanings. In this study, the reliance on transcriptions alone may have limited the depth of the qualitative analysis, as aspects of the verbal and non-verbal communication were not directly observed. The transcription software used may have affected accuracy, particularly with technical terminology or regional accents. The exclusive use of qualitative methods limits the ability to measure the prevalence of specific stressors or benefits. Combining qualitative and quantitative data could provide a more comprehensive view. The study also addresses established technologies like digital radiography, which are now standard. Newer, less studied technologies might deserve more attention.

### 4.6. Practical Implications

Based on the findings of this study and the discussion of digital challenges and opportunities within dental practices, a practical five-stage model (“Framework for Digital Integration in Dental Practices”) has been developed (see Supplement S4). This model outlines the stages of digital adaptation, the associated challenges, and recommended support strategies to facilitate the integration of digital technologies into dental practice workflows. This five-stage framework provides a comprehensive guide for dental practices in navigating the complexities of digital transformation. It identifies the progression from initial awareness to strategic utilisation of digital tools, highlighting both common obstacles and actionable strategies for overcoming them at each stage.

To effectively leverage the benefits of digitalisation in dental practices while minimising potential stressors, the following actions are also recommended:

- Regular, comprehensive training should be provided for all staff, covering both technical skills and strategies for stress management.
- Digital tools should be intuitive, technically reliable, and easy to integrate into daily routines, minimising the risk of additional stress due to complexity.
- Digital systems should be seamlessly incorporated into practice workflows to unlock efficiency gains and reduce workplace strain.
- Management should foster a culture of open communication and provide structured support, including feedback loops and collaborative problem-solving.
- Different employees may require tailored solutions; offering flexible, personalised technical setups can increase acceptance and reduce stress.

### 4.7. Research Implications

This study highlights several key directions for future research on digitalisation in dentistry. Further research focusing on specific digital technologies in dentistry is needed to better understand their individual impact on key factors such as stress, efficiency, and quality of care. Comparative studies evaluating the pros and cons of different digital tools will provide valuable insights into their effectiveness and inform evidence-based decision-making for their integration into clinical practice. Future studies should also examine how individual traits (e.g., age, tech affinity, adaptability) and team dynamics (e.g., leadership, collaboration) influence perceptions of digitalisation and job satisfaction. Research should explore how technological attitudes, willingness to adapt, and training quality affect the successful use of digital tools. While short-term benefits are well documented, the long-term mental health and stress implications of digitalisation require further investigation. Studies should assess whether increasing digitalisation affects long-term job satisfaction, particularly regarding technology-induced stress or reduced interpersonal interaction. The effect of digitalisation may vary based on practice characteristics (e.g., urban/rural, size, digital infrastructure). Comparative studies between different types of practices are recommended. The impact of digital transformation on dentists’ professional identity, role perception, and intrinsic motivation is an emerging area of interest. Future research should compare the effects of various digital tools (e.g., diagnostic vs. communication technologies) on stress perception and job satisfaction.

## 5. Conclusions

The study shows that digital assistance systems have both a positive and negative impact on dentists’ day-to-day work. Benefits such as increased efficiency and error reduction are offset by challenges such as technical faults, training requirements, and increased stress—especially if integration is inadequate. Three factors are crucial for successful implementation: targeted training, technical reliability, and seamless integration into practice processes. Strategic planning and accompanying support are required to maximise the positive effects and minimise stress. Future research should particularly investigate the long-term effects of training measures and the sustainable integration of digital technologies.

## Figures and Tables

**Table 1:** Interview guidelines.

Topic	Category	Subcategory
1: Introduction		
2: Sociodemographic and occupational characteristics of the participants	Practice-specific information data	Practice setup
		Dental laboratory
	Person-specific information	Specialisation/treatment spectrum
		Work schedule
		Professional career
		Employment contract
		Daily work routine
		Age
		Level of education
3: Job demands and job resources in a digital dentistry context	Effects of digital technologies on job demands	Workload Delegation of tasks Susceptibility to errors
		Acceleration
	Effects of digital technologies on job resources	Susceptibility to errors
		Time Work experience
4: Job satisfaction	Effects of digital technologies on work motivation	
	Effects of digital technologies on well-being	
5: Stress perception	Effects of digital technologies on experience of stress	Workload (easing/additional/consistent) Task redistribution/delegation
	Short-term effects of digital technologies on experiences of strain (direct reactions to work-related stressors)	Positive stimulating effects Negative impairing effects (e.g., fatigue, stress)
	Long-term effects of digital technologies (chronic reactions to work-related stressors)	Positive long-term effects Negative long-term effects (e. g. psychosomatic illness, burnout)
6: Support needs and opportunities in digital dentistry	Needs assessment	Support measures
7: Closing		

**Table 2:** Characteristics of the study population.

Category	Characteristic	Frequency (*n*)	Percentage (%)
Gender	Female	12	60
	Male	8	40
Age	20–29	6	30
	30–39	8	40
	40–49	0	0
	50–59	6	30
Position	Assistant dentist	4	20
	Employed dentist	6	30
	Practice owner	10	50
Profession	Approbation	4	20
	Promotion	16	80
Years of work experience	1–9	8	40
	10–19	6	30
	20–29	4	20
	30–39	2	10
Working pensum (%)	0–19	0	0
	20–39	0	0
	40–59	2	10
	60–79	0	0
	80–100	18	90
Federal state	Baden-Württemberg	0	0
	Bavaria	0	0
	Berlin	2	10
	Brandenburg	2	10
	Bremen	0	0
	Hamburg	0	0
	Hesse	6	30
	Mecklenburg Western Pomerania	0	0
	Lower Saxony	0	0
	North Rhine-Westphalia	2	10
	Rhineland Platinate	0	0
	Saarland	0	0
	Saxony	8	40
	Saxony-Anhalt	0	0
	Schleswig Holstein	0	0
	Thuringia	0	0
(Practice) Specialisation	Implantology/Dental surgery	4	20
	Cranio-mandibular dysfunction (CMD)/Functional diagnostics	4	20
	Endodontics/Parodontology	4	20
	General	8	40

**Table 3:** Perceived job demands and resources in a digital dental workplace.

Categories	Job Demands	Job Resources
Work contents and tasks	Workload/overload Working under time pressure Inaccuracies/frequency of errors	Meaning of work Variety of work tasks Susceptibility to errors Acceleration of work processes Simplification of work steps Improvement of results
Work organisation	Adaptation of workflows to practice organisation	Sense of efficiency and productivity Adaptability Delegation of tasks Standardisation of work steps Training/advanced training
Work time	Separation of work and private life	Time saving
Social relations	Comparisons with colleagues Detailed patient education	Exchange between colleagues Communication with dental laboratories Patient feedback Communication within the team
Work equipment	Decision on digital or analogue working method Investment in digital equipment Dependency on technology/technical problems Additional maintenance/repairs	Expansion of treatment options
Work environment	Time-intense screen time	Ergonomic working places Access to patient information regardless of workplace
Personal	Own expectations and emotions Dealing with perfectionism Loss of personal skills Increased controllability	Personal freedom and responsibility Openness to new treatment methods Development of personal physical and mental abilities and skills

**Table 4:** Overview of adverse effects of digital assistance systems on job satisfaction.

Perceived Negative Effects on Job Satisfaction
Dependency on technology
Decline in personal skills
Rapid technological advancement
Lack of control over technology
Increased stress
General workload and bureaucracy

**Table 5:** Overview of positive effects of digital assistance systems on job satisfaction.

Perceived Positive Effects on Job Satisfaction
Work simplification
Motivation enhancement
Sense of fulfilment
Physical and mental health benefits
Skill improvement through experience
Increased economic returns

**Table 6:** Overview of negative effects on perceived stress.

Perceived Negative Effects on Perceived Stress
Uncontrollable malfunctions
Growing dependency on digital systems
Lack of trust in technology
Additional decision-making
Financial pressure
Initial uncertainty
Limited software functionality
Increased coordination and flexibility
Risk of cyberattacks
Pressure of optimisation

## Data Availability

The datasets analysed during the current study are not publicly available due to German national data protection regulations but are available from the corresponding author on reasonable request.

## Supplementary Materials

The following supporting information can be downloaded at: https://www.mdpi.com/article/10.3390/clinpract15050092/s1, Supplement S1: Interview guide; Supplement S2: Analysis of Interview Data Coding; Supplement S3: Additional quotations; Supplement S4: Framework for Digital Integration in Dental Practices: A practical 5-stage model.

## Abbreviations

The following abbreviations are used in this manuscript:

MAXQDA	Max Weber Qualitative Data Analysis
CAD/CAM	Computer-aided design/computer-aided manufacturing
IT	Information technology
LPEK	Local Psychological Ethics Committee
CMD	Cranio-mandibular dysfunction
DVT	Digital volume technology
TMJ	Temporo-mandibular joint
CT	Computed tomography
AI	Artificial intelligence
