# Supplementary material for: Job Demands and Resources Perceived by Dentists in a Digital Dental Workplace and Perceived Effects on Job Satisfaction and Stress: A Qualitative Study
Source: Clin Pract. 2025 May 12;15(5):92. doi: 10.3390/clinpract15050092 (PMC12109974; doi:10.3390/clinpract15050092)
Supplement: Supplementary file 1 [file clinpract-15-00092-s001.zip › Supplement 2_ Codingsystem_Phases.pdf]

Supplement S2: Analysis of Interview Data Coding

Phase 1 – Deductive Category Development Based on the Job Demands-Resources Model (JDR)

Category	Description	Development / Theoretical Basis	Examples of Subcategories
**Job Demands**	Burdens and strains arising from the use of digital technologies in daily dental practice	Initial theoretical category derived from the Job Demands-Resources Model	- Workload<br>- Task delegation<br>- Susceptibility to errors<br>- Acceleration
**Job Resources**	Supportive aspects and potential benefits of digital tools	First derivation of potential resources linked to digitalization	- Time savings<br>- Supportive functions<br>- Work experience
**Job Satisfaction**	Effects of digital technologies on motivation and job satisfaction	Initial differentiation into positive and negative effects	- Motivation<br>- Well-being
**Stress Perception**	Perception of stress related to digital technologies	First distinction between short-term and long-term stress experiences	- Fatigue<br>- Burnout<br>- Positive stimulation

Category	Description	Development / Theoretical Basis	Examples of Subcategories
**Support Needs**	Needs for support in using and implementing digital systems	First exploration of requirements for training and structure	- Training<br>- Technical support

Phase 2 – Deductive-Inductive Analysis of Interview Data

Category	Definition	Development / Differentiation	Key Findings / Sub-Themes
**Job Demands**	Specific job-related demands in digitalized dental practices	Differentiated through recurring statements	- Workload / Overload<br>- Time pressure<br>- Technical problems<br>- Dependency on systems
**Job Resources**	Supporting effects of digital tools	Empirical extension through perceived benefits	- Simplification of work<br>- Improved results<br>- Acceleration<br>- Delegation

Category	Definition	Development / Differentiation	Key Findings / Sub-Themes
**Work Organization**	Organizational adjustments due to digital systems	Newly structured category	- Workflow adaptation<br>- Standardization<br>- Training & upskilling
**Social Relations**	Effects on interpersonal communication	Emerged inductively from the analysis	- Team communication<br>- Patient interaction<br>- Comparison with colleagues
**Work Equipment**	Technological setup and handling	Integration of concrete technical challenges	- System failures<br>- Maintenance needs<br>- Investment requirements
**Personal Factors**	Personal attitudes and mental aspects	Extension to include subjective perceptions and competencies	- Perfectionism<br>- Control<br>- Openness to new methods
**Job Satisfaction**	Impact on satisfaction and motivation	Subjectively perceived effects captured in detail	- Work fulfillment<br>- Skill loss

Category	Definition	Development / Differentiation	Key Findings / Sub-Themes
			- Bureaucracy stress<br>- Economic benefit
**Stress Perception**	Burden due to digital working conditions	Fine differentiation of stress sources	- Cyber risks<br>- Decision-making pressure<br>- Financial strain<br>- Limited software
