# Supplementary material for: Job Demands and Resources Perceived by Dentists in a Digital Dental Workplace and Perceived Effects on Job Satisfaction and Stress: A Qualitative Study
Source: Clin Pract. 2025 May 12;15(5):92. doi: 10.3390/clinpract15050092 (PMC12109974; doi:10.3390/clinpract15050092)
Supplement: Supplementary file 1 [file clinpract-15-00092-s001.zip › Supplement 4_Framework for Digital Integration in Dental Practices A practical 5-stage m.pdf]

Supplement S4

Framework for Digital Integration in Dental Practices: A practical 5-stage model

Stage	Title	Characteristics	Challenges	Recommended Strategies
**1**	**Initial Awareness**	First exposure to digital technologies (e.g., software demonstrations, conferences)	Uncertainty, resistance, lack of orientation	Awareness-building campaigns, showcasing best practice examples, and offering low-barrier entry points.
**2**	**Experimental Use**	Initial application of specific tools (e.g., CAD/CAM, digital radiography)	Error-proneness, cognitive overload, insecurity	Targeted training, mentoring programs, and fostering an open feedback culture.
**3**	**Routine Integration**	Regular use of digital tools; workflows begin to adapt	Coordination issues, partial integration	Workflow restructuring, role clarification, and technical support.
**4**	**Process Optimization**	Digital tools are leveraged to improve efficiency and quality	Outdated systems, stagnation, frustration	Technology management, ongoing education, and timely system updates.
**5**	**Strategic Utilization**	Digitalization is embedded into the practice strategy; the team is confident and proficient	Innovation pressure, diverse individual needs	Customised solutions, a participatory approach, and reflective spaces for continual improvement.
