# Supplementary material for: Job Demands and Resources Perceived by Dentists in a Digital Dental Workplace and Perceived Effects on Job Satisfaction and Stress: A Qualitative Study
Source: Clin Pract. 2025 May 12;15(5):92. doi: 10.3390/clinpract15050092 (PMC12109974; doi:10.3390/clinpract15050092)
Supplement: Supplementary file 1 [file clinpract-15-00092-s001.zip › Supplement1_Interviewguideline_DigitalStress_Gebhardtetal.pdf]

Supplement S1: Interview guide

1. Professional situation

To begin with, I would like to ask you about your professional situation. You are welcome to answer the next questions briefly.

Questions	More specific questions
You work as a dentist. Do you work in a clinic or practice? What is your practice/clinic day like?	• What type of practice/clinic?<br>• What position do you have?
Are you a specialist dentist or do you have a specific focus in your dental work?	
What is your current employment status?	• Employed, private practice?<br>• Full-time, part-time?<br>• Temporary, permanent?<br>• Residency, completed?
How long have you been working as a dentist?	
How long have you been at your current job?	
How many hours do you work per week?	• Do you have the same core working hours every day?<br>• Do you also have weekend or night shifts?
What digital systems is your practice/clinic equipped with?	• Which of these can be found at your treatment chair?

2. Usage behavior and subjective usage evaluation

First of all, I would like to define the digital assistance systems in dental work for you. Digital tools include intraoral scanners, X-ray devices with 3D technology, software solutions and CAD/CAM systems. These can be used in practice organization, diagnostics, treatment planning and clinical implementation.
At the beginning of our conversation, I would like to ask about the use of digital assistance systems. You are then welcome to evaluate them and classify their efficiency with positive or negative experience reports. The questions are very open-ended, so please feel free to say anything you can think of spontaneously. There are no right or wrong answers. Everything that seems important to you in connection with the question is also interesting for me and the study.

Topic	Narrative prompt	More specific questions
Type of digital assistance systems	What types of digital assistance systems do you use for patient	

	care in everyday clinical practice?	
Frequency of use	How often are they used on average?	• Is there a difference between the individual devices in terms of what is used more/less frequently?
Personal experiencen<br><br>*Testimonials/ Subjective evaluation*	How do you perceive the usefulness of digital technologies for patient care?	• What experiences have you had so far?<br>• Positive or negative opinions?<br>• Would you like to see an increase/decrease in the use of digital technologies? Why?

3. Experience of stress and strain

Now that we have talked a lot about your use of digital technologies in everyday practice, we will focus more on the connection between their use and your personal experience of stress and strain.

Topic	Narrative prompt	More specific questions
STRESS	What effects on your workload do you perceive from the use of digital assistance systems (duration, intensity, type of stress)?	• What types of stress do you generally experience as a dentist in your field of work?<br>• To what extent are these influenced by the introduction of new forms of work (in this case: digital systems)?
STRAIN	How does the workload affect your mental stress?	• *Positive stimulating effects: e.g. activation, job satisfaction, motivation, learning effects*<br>• What experiences trigger positive stimulating effects for you?<br>• *Negative effects: e.g. mental fatigue, stress*<br>• What effects do you perceive?

LONG-TERM CONSEQUENCES	What long-term consequences do you expect?	• *Positive: further development of physical and mental abilities and skills, maintaining health, well-being*<br>• *Negative: general psychosomatic disorders/diseases, burnout*
RESSOURCES	Digitalization promises to make work steps easier in many respects. How do you perceive this?	• In which areas can your work be simplified by digital assistance systems?<br>• Can this also speed up work processes?<br>• In your opinion, does this acceleration lead to inaccuracies or an increase in errors?<br>• Can certain work steps be standardized? Could this possibly lead to a reduced susceptibility to errors?
Patient acceptance	What feedback do you get from your patients?	• What positive effects does digitalization have on patient communication?<br>• To what extent does the opinion of patients influence your decision to use conventional or digital treatment methods?
MOTIVATION	What influence do digital resources have on your work motivation?	• Do you feel more motivated to work with modern digital technologies?<br>• What effects do you perceive on your job satisfaction?
WELL-BEING	What influence do digital resources have on your well-being?	• To what extent can digital resources help you to cope better with demands?

4. Needs assessment

The last point I would like to discuss with you is how the use of digital assistance systems can be promoted.

Topic	Narrative prompt	More specific questions
Measures	What measures should be taken to promote the use of digital assistance systems?	• Training and further education opportunities<br>• Further support<br>• Media presence<br>• nformation about advantages and disadvantages, costs and benefits<br>• System design, implementation process
Willingness to learn, openness	How do you feel about dealing with new digital treatment options?	• How willing are you to learn?<br>• How open are you to trying out new treatment methods and changing familiar procedures?<br>• What could increase your interest?
Comparison	Do you feel under pressure to keep up with digital progress?	• Does your practice/clinic advertise modern technology? Why/why not?<br>• Do you know of other practices that invest more in digital equipment? Does this put you under pressure?

5. Socio-demographic data

Finally, I would like to ask you a few general questions about yourself. These serve to make the interviews easier to compare:

- Age:
- Gender:
- Level of education: last (high) school degree, studies
