# Supplementary material for: Job Demands and Resources Perceived by Dentists in a Digital Dental Workplace and Perceived Effects on Job Satisfaction and Stress: A Qualitative Study
Source: Clin Pract. 2025 May 12;15(5):92. doi: 10.3390/clinpract15050092 (PMC12109974; doi:10.3390/clinpract15050092)
Supplement: Supplementary file 1 [file clinpract-15-00092-s001.zip › Supplement3_Additional quotations.pdf]

Supplement S3

Additional quotes

Job Demands

Category	Quote
**Time Pressure**	"I think there's a very high time pressure. You can't really afford to lose a minute." (Participant: female, 20-29 years)
	"The pressure is definitely there. If you stay half an hour longer in a treatment, time gets really tight." (Participant: male, 30-39 years)
**Emotional Demands**	"There are also situations where you're emotionally very challenged, especially with complex patient cases." (Participant: female, 20-29 years)
	"Sometimes patients are emotionally very demanding, and you can definitely feel that." (Participant: female, 30-39 years)
**Physical Demands**	"There are also physical strains from working with equipment or constantly bending down." (Participant: male, 30-39 years)
	"The constant posture during treatment can sometimes be tough." (Participant: male, 30-39 years)

Job Ressources

Category	Quote
**Social Support**	"Support from colleagues is very important to me, especially when something unexpected happens." (Participant: male, 30-39 years)
	"I always feel like I can rely on my colleagues, which takes a lot of stress off me." (Participant: female, 20-29 years)
**Autonomy**	"I appreciate when I can make decisions independently, without always having to wait for approval." (Participant: female, 30-39 years)

Category	Quote
	"The freedom to manage the treatment process myself is a great resource for me." (Participant: female, 30-39 years)
**Professional Development**	"The opportunity for professional development and training motivates me a lot." (Participant: male, 30-39 years)
	"I consider further training very valuable because it helps me expand my skills." (Participant: female, 30-39 years)
**Technological Support**	"Technology makes many things easier for me, especially in managing and documenting patient data." (Participant: male, 30-39 years)
	"The digital systems allow for better planning and execution of treatments." (Participant: female, 20-29 years)
**Time Resources**	"With the digital tools, I have less effort and can work faster." (Participant: female, 20-29 years)
	"The automation of certain processes helps me save time and be under less pressure." (Participant: female, 30-39 years)
**Motivation Enhancement**	"The intraoral scanner is definitely the most exciting of all the things, but overall, I can say I'm highly motivated to keep going with it." (participant: male, age 50-59)
	"Yes, it's definitely something new. And in general, something new is potentially also something that makes you more motivated. I think you're more motivated in that sense because you're thinking about what else can be done." (participant: female, age 20-29)
**Sense of Fulfillment**	"Or I place implants in three dimensions, and afterwards they fit perfectly, making the patient happy—and me too." (participant:female, age 50-59)
	"I'm happy when I get to sit in the lab and make the crown myself." (participant: female, age 20-29)

Category	Quote
**Physical and Mental Health Benefits**	"Absolutely not. I can't see any reason why I would get burnout because of digitalization. It actually helps me more." (participant: female, age 50-59)
**Skill Improvement through Experience**	"Exactly, it will definitely get better at some point. That you'll be in sync with the feeling, I would say." (participant: female, age 30-39)
	"No, I think it's important to sometimes make mistakes or not do something perfectly, so that you have the drive to improve next time. I believe it's all part of the process." (participant: female, age 20-29)
	"The younger employees are all 'digital natives,' and they can work with these tools in a way that differs greatly from those who are more established." (participant: male, age 30-39)
**Increased Economic Returns**	"Yes, but when you eventually realize that everything is working well and fitting perfectly, it's a wonderful feeling. Especially when you know that you created it yourself." (participant: female, age 20-29)
	"I think that overall it will get better, especially if many of the things I'm hoping for now work well. Then I believe it will bring ease and, with that, greater satisfaction." (participant: female, age 30-39)

Category	Quote
**Negative Effects on Stress Experience**	"That causes stress. So, when it doesn't work. When it does work, it's great." (participant: male, age 30-39)
	"If the software doesn't work for some reason, then even the best sterilizer won't help you." (participant: male, age 30-39)
	"What's important is that everything becomes a bit more precise, especially regarding planning. That the uncertainty factor—whether it's about an implant drilling template—needs to be clear." (participant: female, age 20-29)
	"The only thing […] is when you have external disturbances that I cannot control. For example, if the scanner collapses in the middle of a scan for some reason." (participant: female, age 50-59)
	"Well, the increasing dependence on digital devices often brings the problem of what to do when the first device doesn't work." (participant: male, age 30-39)
	"If I had negative experiences and faced setbacks every day—if things didn't fit, if it didn't look nice, if the height wasn't right, if the shape wasn't right—then that would be something else." (participant: female, age 50-59)
	"It's perhaps important to mention that it's a higher initial investment. And that cost pressure is definitely present at the beginning because you don't really know how it will turn out." (participant:, male, age 50-59)
	"If the program constantly crashes, it's naturally frustrating." (participant: male, age 30-39)
	"What really stresses me out mentally and honestly drives me crazy is when software takes forever to process." (participant: male, age 30-39)

Category	Quote
	"But you also need to know the limits. As I said, there are definitely things that you just can't do digitally, unfortunately." (participant: female, age 50-59)<br><br>"Well, basically, as we mentioned earlier, when I want to use the scanner, and someone else is using it, that already impacts my work because I can't move forward." (participant: female, age 20-29)<br><br>"The worst thing that could happen, of course, would be a hacker attack or something like that." (participant: female, age 20-29)<br><br>"I often worry about whether it was perfect, and if it wasn't, what caused it and what I could have done better." (participant: female, age 20-29)
**Positive Effects on Stress Experience**	"No, not really. I don't let it stress me out." (participant: female, age 30-39)<br><br>"You can experiment a lot and say, 'Let's try it this way,' or 'Let's give this a try,' or 'Let's design it differently this time.'" (participant: female, age 30-39)<br><br>"You eventually lose track if you don't keep up. You're forced to stay engaged; otherwise, you won't know how it works." (participant: male, age 30-39)<br><br>"But I do hope that we continue to stay progressive or even become more advanced and that we keep learning new things." (participant: female, age 20-29)<br><br>"Yes, I have the ambition to continue developing myself." (participant: female, age 30-39)<br><br>"I'm very technically inclined and have a strong affinity for software by nature." (participant: male, age 30-39)

Category	Quote
	"Yes, improving oneself, but also discussing things with others. You also learn from that—how others treat and plan." (participant: female, age 20-29)
**Negative Effects on Stress Experience**	
**Uncontrollable Malfunctions (e.g. Software Issues)**	"It's annoying that when it doesn't work, you end up spending more time than usual." (Participant: female, 50-59 years)
	"That causes stress. So, when it doesn't work. When it does work, it's great." (Participant: male, 30-39 years)
	"If the software doesn't work for some reason, then even the best sterilizer won't help." (Participant: male, 30-39 years)
	"Or what can also happen sometimes is that you have the device, and it freezes, and you don't know why. And everything you scanned suddenly disappears." (Participant: female, 20-29 years)
**Dependence on Technology**	"The only thing […] is when you have external disturbances that I cannot control. For example, if the scanner collapses in the middle of a scan for some reason." (Participant: female, 50-59 years)
	"Well, the increasing dependence on digital devices often brings the problem of what to do when the first device doesn't work." (Participant: male, 30-39 years)
**Lack of Trust in Technology**	"If I had negative experiences and faced setbacks every day—if things didn't fit, if it didn't look nice, if the height wasn't right, if the shape wasn't right—then that would be something else." (Participant: female, 50-59 years)
	"What's important is that everything becomes a bit more precise, especially regarding planning. That the uncertainty

Category	Quote
	factor—whether it’s about an implant drilling template—needs to be clear." (Participant: female, 20-29 years)
**Decision Pressure**	"You suddenly face the decision before treating a patient for whom you want to place an implant: should I use a template or not?" (Participant: female, 20-29 years)
**Financial and Technical Uncertainties**	"It’s perhaps important to mention that it’s a higher initial investment. And that cost pressure is definitely present at the beginning because you don’t really know how it will turn out." (Participant: male, 50-59 years)
	"If the program constantly crashes, it’s naturally frustrating." (Participant: male, 30-39 years)
	"What really stresses me out mentally and honestly drives me crazy is when software takes forever to process." (Participant: male, 30-39 years)
	"But you also need to know the limits. As I said, there are definitely things that you just can't do digitally, unfortunately." (Participant: female, 50-59 years)
**Coordination and Flexibility**	"Well, basically, as we mentioned earlier, when I want to use the scanner, and someone else is using it, that already impacts my work because I can’t move forward." (Participant: female, 20-29 years)
**Cybersecurity Risks**	"The worst thing that could happen, of course, would be a hacker attack or something like that." (Participant: female, 20-29 years)
**Perfectionism Pressure**	"I do take a lot of things home with me. I often worry about whether it was perfect, and if it wasn’t, what caused it and what I could have done better." (Participant: female, 20-29 years)
	"Yes, you often end up comparing yourself as well." (Participant: female 20-29 years)

Category	Quote
**Positive Effects on Stress Experience**	
**Motivation and Positive Attitude**	"Fatigue and stress aren’t an issue for me at all. So, more positive overall. And if there’s something I don’t know, then of course you’re a bit behind." (Participant: female, 50-59 years)
	"So it definitely has a psychologically positive effect, you could say." (Participant: female, 30-39 years)
	"No, not really. I don’t let it stress me out." (Participant: female, 30-39 years)
**Flexibility and New Possibilities**	"Of course, it’s a lot more fun. You can also do so much more with digitalization. Especially in the lab, we have completely different possibilities with digital work." (Participant: female, 30-39 years)
	"You can experiment a lot and say, 'Let’s try it this way,' or 'Let’s give this a try,' or 'Let’s design it differently this time.' It’s definitely a challenge, but one that’s also fun." (Participant: female, 30-39 years)
**Learning and Development**	"Yes, digital work absolutely motivates me, and I’m definitely willing to acquire new skills to push the technical possibilities even further." (Participant: male, 30-39 years)
	"Mentally, at least, the constant contact with digital media forces you to stay up-to-date. So, in that sense, it's definitely progress." (Participant male: 30-39 years)
	"You eventually lose track if you don’t keep up. You’re forced to stay engaged; otherwise, you won’t know how it works." (Participant: male, 30-39 years)

Category	Quote
**Ambition and Striving for Perfection**	"Yes, I have the ambition to continue developing myself. […] You really have to make sure that it works and that it is definitely improved to be effective and good." (Participant: female, 30-39 years)
**Precision and Efficiency**	"And I definitely notice it, thanks to the good fit and advanced technology. The collaboration with the technician has improved significantly." (Participant: male, 50-59 years)
	"For example, when placing an implant, it fits exactly as planned, and that's a great feeling." (Participant: female, 30-39 years)
**Safety and Reliability**	"Well, for example, when placing an implant, it fits exactly as planned, and that's a great feeling. The peace of mind knowing you won't be losing sleep over possibly injuring a nerve is very reassuring." (Participant: female, 30-39 years)
**Feedback and Collaboration**	"I'm very technically inclined and have a strong affinity for software. I definitely find it much more appealing to do a 3D scan, as it's such an interesting process compared to taking an impression, where I have to deal with messy materials in the patient's mouth." (Participant: male, 30-39 years)
	"It means it's also a source for sharing experiences. Discussing patient cases becomes easier through digitalization, as everyone can access the information at any time." (Participant: female, 20-29 years)
	"Yes, improving oneself, but also discussing things with others. You also learn from that—how others treat and plan." (Participant: female, 20-29 years)
